# Sex-based differences in the association of resistance training levels with the risk of hypertension

**DOI:** 10.3389/fpubh.2024.1401254

**Published:** 2024-06-06

**Authors:** Jae Ho Park, Hyun-Young Park

**Affiliations:** ^1^Division of Population Health Research, Department of Precision Medicine, Korea National Institute of Health, Korea Disease Control and Prevention Agency, Cheongju, Republic of Korea; ^2^Korea National Institute of Health, Korea Disease Control and Prevention Agency, Cheongju, Republic of Korea

**Keywords:** hypertension, exercise, resistance training, sex differences, population study

## Abstract

**Introduction:**

Hypertension is a primary risk factor for cardiovascular disease and all-cause mortality. This study investigated sex-based differences in the association between the risk of hypertension and resistance training (RT) levels, including training frequency and period.

**Methods:**

We enrolled 162,102 participants from nationwide Korean cohorts. The training period (months) and frequency (per week) of RT were used to investigate the presence of an inverse dose–response relationship between RT levels and the risk of hypertension. Multiple logistic regression models were used to evaluate the risk of hypertension in relation to RT levels.

**Results:**

The prevalence of hypertension in the study population was 36.28% in men and 26.94% in women. Performing RT was associated with an 8% reduction in the risk of hypertension in women but not in men. In women, performing RT for 3–4 days/week, compared with not performing RT, reduced the risk of hypertension by 11%, even after adjusting for covariates, including RT time per week and period. However, in men, no significant association was observed between training frequency and the risk of hypertension. We also evaluated the risk of hypertension by simultaneously considering both the RT frequency and period. Performing RT for 3–4 days/week and ≥5 days/week were markedly related to 14 and 11% hypertension risk reduction, respectively, in women who had been performing RT for at least 6 months.

**Conclusion:**

Given that no inverse dose–response association was observed between RT frequency and hypertension risk, engaging in RT for 3–4 days/week for at least 6 months is recommended for women. Further longitudinal studies are needed to verify sex-based differences in the antihypertensive effects of regular RT.

## 1 Introduction

Hypertension or high blood pressure (BP) is a primary risk factor for cardiovascular disease (CVD) and all-cause mortality (1, 2). According to the World Health Organization (WHO), the prevalence of hypertension has increased globally, and an estimated 1.4 billion people, almost one-fifth of the world’s population, suffer from hypertension (3). In Korea, one of the fastest-aging countries in the world, the prevalence of hypertension among the older adult has increased from 62 to 66% in women and from 49 to 59% in men over the last decade (4). Consequently, increasing interest in preventive and therapeutic strategies for hypertension has been observed.

Recent hypertension guidelines recommend participating in moderate-intensity aerobic exercise training for at least 150 min per week to prevent and/or manage hypertension (5, 6). In recent meta-analyses, aerobic exercise training effectively reduced both systolic BP (SBP) and diastolic BP (DBP) in patients with hypertension (7, 8). Current guidelines also recommend performing resistance training (RT), which causes the major muscle groups in the body to work against external resistance, for 2–3 days per week to improve musculoskeletal fitness, blood glucose levels, insulin sensitivity, and BP (5, 6, 9). However, unlike aerobic exercise, the antihypertensive effects of RT remain unclear and controversial. Recent meta-analysis has shown that regular RT significantly reduces SBP and DBP in both individuals with prehypertension and hypertension (10). However, in another meta-analysis, significant BP-lowering effects were observed in participants who performed RT for 3 times per week compared with RT 2 times per week, and only moderate-intensity RT among other training intensities, including low- and high-intensity, was effective in reducing both SBP and DBP (11). Several randomized controlled trials (RCTs) have also reported no significant BP-lowering effects after 8 weeks of progressive (from low- to moderate-intensity) RT for 2 days per week (12), 13 weeks of moderate-intensity RT for 3 days per week (13), and 8 weeks of high-intensity RT for 3 days per week (14). Therefore, the BP-lowering effects of RT have been reported to be inconsistently dependent on training variables, including RT frequency and intensity. A few epidemiological studies have investigated the association between regular RT and the risk of hypertension beyond its BP-lowering effect. Although a recent cohort study revealed that regular RT for more than 1 day per week was associated with a significant hypertension risk reduction (15), this study did not consider a potential sex-based difference in the association between the variables and the presence of an inverse graded dose–response association according to RT levels, such as training frequency and period.

In the United States, the proportion of adults who perform RT for 2 days or more per week has increased by 5.4% among men (from 25.7 to 31.1%) and 6% among women (from 18.3 to 24.3%) over the past decade (16). As the sex gap in RT participation rates has reduced, a growing interest has been observed in the investigation of sex-based differences in the antihypertensive effect of RT. However, to the best of our knowledge, few studies have investigated these sex-based differences. Recent meta-analytical evidence has reported that regular RT significantly reduces both SBP and DBP in women but not in men (11). According to a longitudinal study, including RT in the physical activity (PA) schedule of participants who met the current PA guideline (≥150 min/week of moderate-intensity PA) further reduced the risk of incident hypertension by 35% in women but not in men (17). In contrast, higher levels of muscular strength, a major outcome of long-term RT, are markedly associated with a reduced risk of hypertension in both sexes (18, 19). Considering these contradictory results, further studies are necessary to investigate sex-based differences in the antihypertensive effects of RT. In particular, further research on specific RT levels, such as training frequency and period, for an antihypertensive effect according to sex is needed to provide sex-specific recommendations.

Therefore, the purpose of the present study was to investigate the association between RT regularity and hypertension risk reduction using data from large nationwide cohorts in Korea. We further aimed to examine the presence of an inversely graded dose–response relationship between RT levels (i.e., training frequency and period) and the risk of hypertension.

## 2 Materials and methods

### 2.1 Study participants

The present study used data from the Korean Genome and Epidemiology Study (KoGES), conducted by the Korea National Institute of Health. The KoGES is a consortium project consisting of 6 prospective cohort studies and aims to investigate the environmental and genetic etiologies of non-communicable chronic diseases in Korea, including hypertension, diabetes mellitus, CVD, and cancer (20). In this study, we used 2003–2013 data from the KoGES Health Examinee study, which included 173,202 urban residents aged 40–79 years, the KoGES Cardiovascular Disease Association study, which included 28,337 rural residents aged 40–91 years, and the fourth wave of the KoGES Ansan and Ansung study conducted in 2007–2008, which included 6,688 participants aged 44–76 years who lived in Ansan (an urban area) and Ansung (a rural area). All participants underwent face-to-face surveys and physical examinations were conducted by trained medical staff. A detailed description of these cohort studies has been provided previously (20).

**Figure 1:**
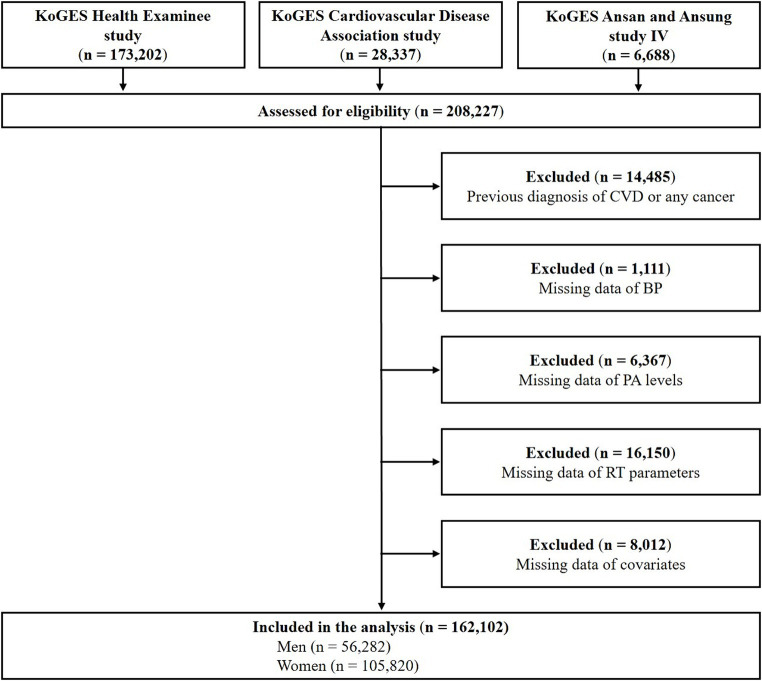
Flow diagram of participant inclusion and exclusion. CVD, cardiovascular disease; BP, blood pressure; PA, physical activity; RT, resistance training.

Among the 208,227 participants from the 3 cohorts, the following were excluded from this study: those with a clinical history of CVD and any type of cancer (*n* = 14,485), those without data on BP (*n* = 1,111), those without data on PA levels (*n* = 6,367), those without data on RT parameters (*n* = 16,150), and those without data on covariates (*n* = 8,012). A total of 162,102 participants (105,820 women) were included in the final analysis (Figure 1). This study was approved by the Institutional Review Board of the Korea National Institute of Health, Korea Disease Control and Prevention Agency (Approval No. KDCA-2024-02-12-P-01).

### 2.2 Measurement of RT levels

**Figure 2:**
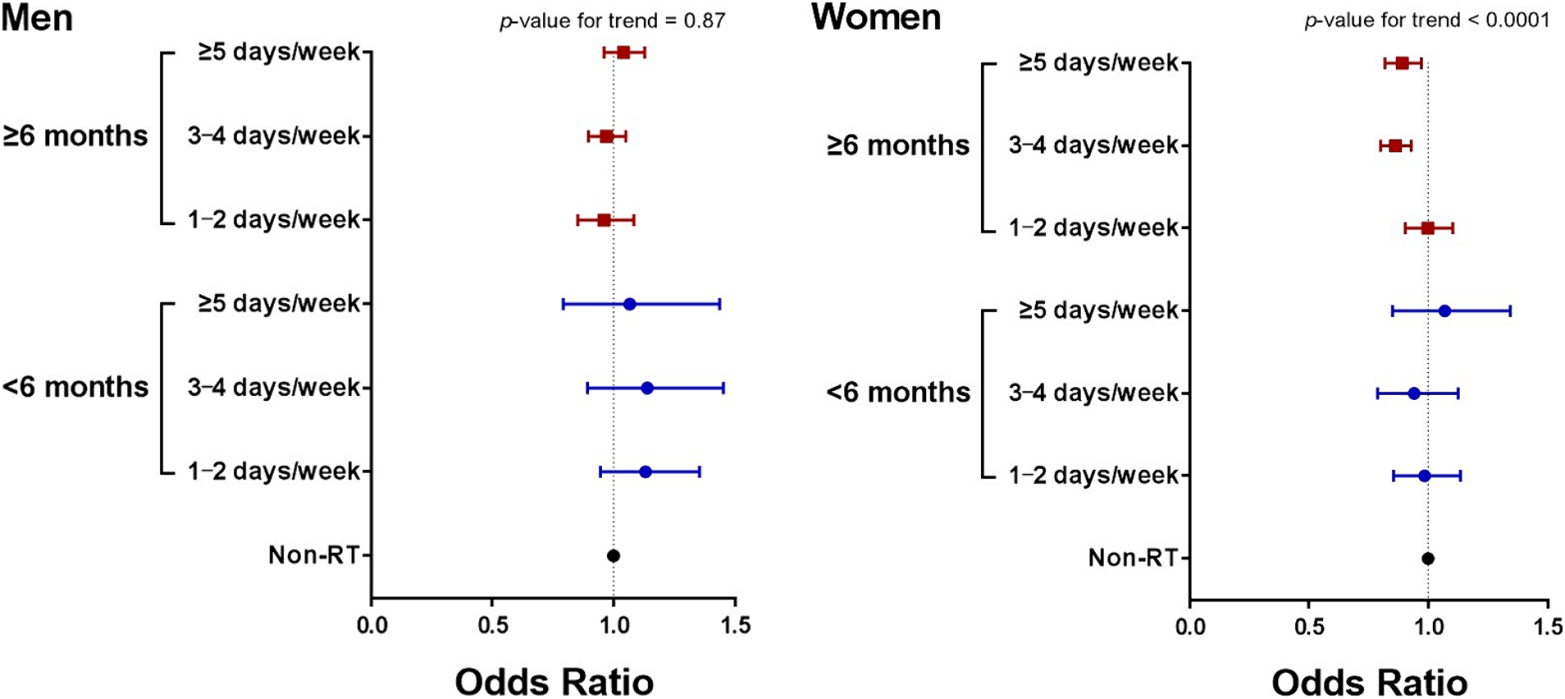
Odds ratios for hypertension prevalence according to period and frequency of RT. Adjusted for age, drinking, smoking, educational level, BMI, T-Chol, eGFR, PA time, and diabetes mellitus. RT, resistance training; BMI, body mass index; T-Chol, total cholesterol; eGFR, estimated glomerular filtration rate; PA, physical activity.

All participants completed questionnaires regarding details of their RT levels. RT was defined as any training program involving muscle contraction against external resistance such as body weight, weight machines, barbells, and dumbbells. The frequency (per week), training time (min/week), and training period (months) of RT were assessed to examine RT levels. Regular RT was defined as participating in an RT program for more than 1 day per week. Participants were divided into 2 groups based on the regularity of RT: “non-RT (not performing RT)” and “RT (performing RT).” To investigate the presence of an inversely graded dose–response relationship between RT levels and the risk of hypertension, the training period and frequency of RT were analyzed. Based on the frequency of RT, participants were categorized into one of the following 4 subgroups: “non-RT (not performing RT),” “1–2 days/week,” “3–4 days/week,” and “≥5 days/week.” Based on the RT training period, participants were also classified into one of the following 3 subgroups: “non-RT (not performing RT),” “<6 months,” and “≥6 months.” Finally, as shown in Figure 2, the participants were divided into one of the 7 groups indicated above by simultaneously considering the training period and frequency of RT.

### 2.3 Definition of hypertension

Hypertension was defined based on a previous diagnosis by a physician, current use of antihypertensive drugs, SBP ≥140 mmHg, or DBP ≥90 mmHg. Trained healthcare providers measured BPs using standard methods. SBP and DBP were obtained by averaging two readings from the arm with the highest SBP after the participant had rested for 5 min in a seated position.

### 2.4 Covariates

Sociodemographic and health-related factors, including age, sex, educational level, drinking and smoking habits, PA time, body mass index (BMI), waist circumference (WC), diabetes mellitus status, and laboratory parameters, were included in our analyses. Educational level was divided into elementary school graduates or lower, middle or high school graduates, and college graduates or higher. Drinking and smoking habits were classified as “never,” “former,” and “current.” PA time was defined as the total time (min/week) spent engaging in moderate-intensity leisure-time PA in a typical week. Moderate-intensity leisure-time PA was defined as participation in sports or engagement in exercises that resulted in sweating.

Anthropometric data including height, body weight, and WC were measured by trained healthcare providers using standardized methods. BMI was calculated as body weight (kg) divided by height (m) squared (kg/m^2^). Blood samples were collected after an overnight fasting period of 8 h. Biochemical assays were performed to determine the total cholesterol (T-Chol), high-density lipoprotein cholesterol (HDL-C), triglyceride (TG), fasting blood glucose (FBG), and creatinine levels. The estimated glomerular filtration rate (eGFR) was calculated using the following formula, with creatinine expressed in mg/dL (21): eGFR (mL/min per 1.73 m^2^) = 175 × (creatinine)^–1.154^ × (age)^–0.203^ × (0.742, if female). Diabetes mellitus was defined based on a previous diagnosis by a physician, current use of antidiabetic medications, including insulin and oral hypoglycemic agents, FBG ≥126 mg/dL, or glycated hemoglobin ≥6.5%. Detailed information on the biochemical analyses is described in a previous study (20).

### 2.5 Statistical analysis

All statistical analyses were conducted using SAS software (version 9.4; SAS Institute, Cary, North Carolina, United States). Participant characteristics are presented as descriptive statistics. Continuous variables are presented as mean ± standard deviation, whereas categorical variables are expressed as absolute frequencies and percentages (%). The chi-squared test was used to determine intergroup differences in educational level, drinking and smoking habits, RT regularity, and prevalence of hypertension and diabetes mellitus. Independent *t*-tests were used to compare age, PA time, BMI, WC, SBP, DBP, T-Chol, HDL-C, TG, FBG, creatinine, and eGFR between the groups.

Multiple logistic regression models were used to evaluate the odds ratios (ORs) and 95% confidence intervals (CIs) for hypertension prevalence. The models were adjusted for age, drinking, smoking, educational level, BMI, T-Chol level, eGFR, PA time, RT time (min/week), RT period (months), and diabetes mellitus status. Subgroup analyses were performed for each sex to investigate the association between the risk of hypertension and performance of long-term RT (≥6 months) for 3 or more days/week, considering age (<65 and ≥65 years), educational level (≤middle school and ≥high school), current drinking habits (no and yes), smoking status (never and ever), BMI (<25 and ≥25 kg/m^2^), and diabetes mellitus status (no and yes). The *p*-value for the interaction was estimated to assess the consistency of the associations across the subgroups. All tests were two-tailed, and statistical significance was set at a *p*-value < 0.05.

## 3 Results

A total of 162,102 participants (105,820 women) were included in the analysis. Table 1 shows the characteristics of the study participants based on RT regularity and sex. The proportions of men and women engaging in regular RT were 15.84 and 13.84%, respectively. In women, the prevalence of hypertension was significantly lower in the RT group than in the non-RT group, whereas in men, no significant difference in the prevalence of hypertension was observed. In both sexes, the RT group showed markedly lower mean age, WC, TG, and FBG and lower proportions of never drinkers, current smokers, and patients with diabetes mellitus than the non-RT group. In contrast, the RT group exhibited significantly higher PA time, HDL-C, and a higher proportion of individuals with a high educational level (≥college) than the non-RT group. In men, compared with the non-RT group, the RT group was significantly associated with higher BMI and creatinine levels but lower eGFR. Women in the RT group had a markedly lower BMI but higher eGFR than women in the non-RT group. SBP, DBP, and T-Chol levels were significantly lower in the RT group than in the non-RT group in women but not in men.

**Table 1:** Characteristics of study participants based on RT regularity and sex.

Variables	Men (*n* = 56,282)		*p*-value	Women (*n* = 105,820)		*p*-value
	non-RT (*n* = 47,365)	RT (*n* = 8,917)		non-RT (*n* = 91,172)	RT (*n* = 14,648)	
**Age** (years)	54.07 ± 8.81	53.05 ± 8.38	<0.0001	53.02 ± 8.23	50.85 ± 7.46	<0.0001
**Educational level**, n (%)			<0.0001			<0.0001
≤Elementary school	6,810 (14.38)	493 (5.53)		23,848 (26.16)	1,665 (11.37)	
Middle/high school	25,535 (53.91)	4,472 (50.15)		52,621 (57.71)	9,171 (62.61)	
≥College	15,020 (31.71)	3,952 (44.32)		14,703 (16.13)	3,812 (26.02)	
**Drinking habit**, n (%)			<0.0001			<0.0001
Never drinker	9,733 (20.55)	1,501 (16.83)		61,420 (67.37)	8,970 (61.24)	
Ex-drinker	3,188 (6.73)	608 (6.82)		1,776 (1.95)	353 (2.41)	
Current drinker	34,444 (72.72)	6,808 (76.35)		27,976 (30.68)	5,325 (36.35)	
**Smoking habit**, n (%)			<0.0001			<0.0001
Never smoker	12,993 (27.43)	2,595 (29.10)		87,831 (96.33)	14,203 (96.96)	
Ex-smoker	17,779 (37.54)	4,095 (45.92)		1,109 (1.22)	202 (1.38)	
Current smoker	16,593 (35.03)	2,227 (24.98)		2,232 (2.45)	243 (1.66)	
**PA time** (min/week)	144.04 ± 226.12	284.59 ± 248.01	<0.0001	117.51 ± 193.93	265.24 ± 227.03	<0.0001
**BMI** (kg/m^2^)	24.33 ± 2.81	24.58 ± 2.58	<0.0001	23.82 ± 3.03	23.34 ± 2.71	<0.0001
**WC** (cm)	85.84 ± 7.65	85.50 ± 7.19	<0.0001	79.10 ± 8.42	77.17 ± 7.62	< 0.0001
**SBP** (mmHg)	125.85 ± 15.11	125.84 ± 14.75	0.93	121.54 ± 15.94	118.79 ± 15.18	< 0.0001
**DBP** (mmHg)	79.10 ± 10.04	79.30 ± 9.89	0.08	75.31 ± 10.07	74.03 ± 9.82	<0.0001
**T-Chol** (mg/dL)	194.48 ± 34.80	194.69 ± 33.10	0.58	200.48 ± 35.94	198.14 ± 34.91	<0.0001
**HDL-C** (mg/dL)	48.91 ± 12.03	50.61 ± 11.89	<0.0001	55.18 ± 12.88	58.28 ± 13.21	<0.0001
**TG** (mg/dL)	155.56 ± 112.19	146.54 ± 105.41	<0.0001	117.31 ± 77.66	103.97 ± 64.83	<0.0001
**FBG** (mg/dL)	99.67 ± 25.69	97.75 ± 21.29	<0.0001	93.41 ± 20.04	91.24 ± 16.06	<0.0001
**Creatinine** (mg/dL)	0.98 ± 0.22	1.00 ± 0.19	<0.0001	0.75 ± 0.18	0.75 ± 0.12	0.08
**eGFR** (ml/min per 1.73 m^2^)	82.59 ± 14.70	80.91 ± 13.32	<0.0001	84.19 ± 16.03	84.52 ± 14.90	<0.05
**Diabetes mellitus**, n (%)	6,210 (13.11)	1,025 (11.49)	<0.0001	7,263 (7.97)	798 (5.45)	<0.0001
**Hypertension**, n (%)	17,203 (36.32)	3,215 (36.05)	0.63	25,389 (27.85)	3,116 (21.27)	<0.0001

RT, resistance training; PA time, total time of regular participation in any sport or exercise to the point of sweating; BMI, body mass index; WC, waist circumference; SBP, systolic blood pressure; DBP, diastolic blood pressure; T-Chol, total cholesterol; HDL-C, high-density lipoprotein cholesterol; TG, triglyceride; FBG, fasting blood glucose; eGFR, estimated glomerular filtration rate.

The characteristics of the study participants based on hypertension status and sex are shown in Supplementary Table 1. The prevalence of hypertension in our study population was 36.28 and 26.94% in men and women, respectively. In both sexes, the hypertension group compared with the normotensive group was significantly associated with higher mean age, PA time, BMI, WC, TG, FBG, creatinine, and prevalence of diabetes mellitus, but lower HDL-C, eGFR, and proportion of a high educational level (≥college), and current smokers. Among men, the hypertension group showed a markedly higher prevalence of current drinkers than the normotensive group. In women, compared with the normotensive group, the hypertension group had significantly lower proportions of current drinkers and individuals engaging in regular RT, but higher T-Chol levels.

Table 2 shows the association between RT regularity and risk of hypertension after adjusting for covariates. Men had a significantly longer training time (*p* < 0.01) and period (*p* < 0.0001), as well as a markedly higher training frequency (*p* < 0.0001) and rate of a long-term RT program (≥6 months; *p* < 0.0001) than women. However, performing RT was associated with an 8% reduction in hypertension risk in women (*p* < 0.001) but not in men. We further investigated the presence of an inverse dose–response association between RT levels and the risk of hypertension. As shown in Table 3, no inverse dose–response relationship was observed between RT frequency and the risk of hypertension in either sex. In women, compared with those who did not engage in RT, performing RT for 3–4 days/week decreased the risk of hypertension by 11% after adjusting for covariates (*p* < 0.05). However, in men, no significant association was observed between training frequency and the risk of hypertension.

**Table 2:** Odds ratios for hypertension prevalence according to RT regularity and sex.

	*N*	RT levels				Crude model OR (95% CI)	Adjusted model OR (95% CI)
		Frequency	Time	Training period			
		(days/week)	(min/week)	(month)	≥6 month (%)		
**Men**							
non-RT	47,365	–	–	–	–	1 (reference)	1 (reference)
RT	8,917	3.99 ± 1.87^b^	230.20 ± 186.76^a^	19.94 ± 39.57^b^	87.45^b^	0.99 (0.94–1.04)	1.01 (0.96–1.07)
**Women**							
non-RT	91,172	–	–	–	–	1 (reference)	1 (reference)
RT	14,648	3.59 ± 1.67^b^	223.58 ± 163.94^a^	14.17 ± 21.04^b^	80.80^b^	0.70 (0.67–0.73)^****^	0.92 (0.88–0.96)^***^

RT, resistance training; OR, odds ratio; CI, confidence interval; BMI, body mass index; T-Chol, total cholesterol; eGFR, estimated glomerular filtration rate; PA, physical activity; ^a^*p* < 0.01 compared women with men in RT group; ^b^*p* < 0.0001 compared women with men in RT group; ^***^p < 0.001; ^****^p < 0.0001; Adjusted for age, drinking, smoking, educational level, BMI, T-Chol, eGFR, PA time, and diabetes mellitus.

**Table 3:** Odds ratios for hypertension prevalence according to RT frequency and sex.

	*N*	RT levels				Crude model OR (95% CI)	Adjusted model OR (95% CI)
		Frequency	Time	Training period			
		(days/week)	(min/week)	(month)	≥6 month (%)		
**Men**							
non-RT	47,365	0.00 ± 0.00	0.00 ± 0.00	0.00 ± 0.00	0.00	1 (reference)	1 (reference)
1–2 days/week	1,990	1.54 ± 0.50	76.58 ± 60.51	17.14 ± 36.35	70.60	0.87 (0.79–0.96)^**^	1.07 (0.96–1.19)
3–4 days/week	3,599	3.44 ± 0.50	204.17 ± 118.58	20.92 ± 39.82	91.03	0.92 (0.86–0.99)^*^	1.06 (0.96–1.17)
≥5 days/week	3,328	6.05 ± 0.89	350.21 ± 216.38	20.55 ± 41.06	93.66	1.15 (1.07–1.23)^***^	1.12 (0.98–1.27)
**Women**							
non-RT	91,172	0.00 ± 0.00	0.00 ± 0.00	0.00 ± 0.00	0.00	1 (reference)^a^	1 (reference)
1–2 days/week	4,018	1.65 ± 0.48	96.12 ± 60.47	10.85 ± 14.74	65.38	0.74 (0.68–0.79)^****^	1.01 (0.92–1.10)
3–4 days/week	6,399	3.37 ± 0.48	209.06 ± 101.81	14.87 ± 20.98	85.47	0.63 (0.59–0.67)^****^	0.89 (0.80–0.98)^*^
≥5 days/week	4,231	5.75 ± 0.86	366.58 ± 194.19	16.26 ± 25.40	88.40	0.78 (0.72–0.84)^****^	0.93 (0.80–1.07)

RT, resistance training; OR, odds ratio; CI, confidence interval; BMI, body mass index; T-Chol, total cholesterol; eGFR, estimated glomerular filtration rate; PA, physical activity; ^a^*p* < 0.0001 in the test for trend of ORs; ^*^p < 0.05; ^**^p < 0.01; ^***^p < 0.001; ^****^p < 0.0001; Adjusted for age, drinking, smoking, educational level, BMI, T-Chol, eGFR, PA time, RT time, RT period, and diabetes mellitus.

Figure 2 presents results on analysis the risk of hypertension evaluated by simultaneously considering both the training frequency and period of RT after adjustment for covariates. Among individuals who performed RT for less than 6 months, no significant associations were observed between training frequency and the risk of hypertension, regardless of sex. Among female participants who performed RT for 6 or more months, performing RT for 3–4 days/week and ≥5 days/week were related to a risk reduction of 14% (*p* < 0.0001) and 1% (*p* < 0.01), respectively, compared with their counterparts who did not perform RT. In men, however, no significant associations between training frequency and the risk of hypertension was observed, regardless of whether RT was performed for 6 or more months.

Subgroup analyses were performed for each sex to investigate whether the association between hypertension risk reduction and the performance of long-term RT (≥6 months) for 3 or more days/week was consistent in the various subgroups, including age, educational level, current drinking habits, smoking status, BMI, and diabetes mellitus status. In men, no significant relationship between long-term RT and a reduced risk of hypertension was observed in any subgroup (Supplementary Table 2). In women, the significance of the association between long-term RT and hypertension risk reduction was different in some of the subgroups (Supplementary Table 3). Particularly, the protective benefit of long-term RT against hypertension was significant only in those who were <65 years (*p* < 0.0001), had never smoked (*p* < 0.0001), and with a BMI <25 kg/m^2^ (*p* < 0.0001). Although a significant interaction was observed for current drinking habits (*p* for interaction < 0.001), long-term RT conferred a protective benefit against hypertension in both subgroups (no and yes).

## 4 Discussion

To the best of our knowledge, the present study is the first to examine sex-based differences in the association between the risk of hypertension and specific RT levels (e.g., training frequency and period) in nationwide Korean cohorts. This study indicated no inverse dose–response association between RT levels and the risk of hypertension in either sex. When RT was performed for at least 6 months, an RT frequency of 3–4 days/week and ≥5 days/week in women were markedly related to 14 and 11% hypertension risk reduction, respectively. Taken together, given that no inverse dose–response association was observed between RT frequency and hypertension risk, we recommend that women should perform RT for 3–4 days/week for at least 6 months. Moreover, considering that RT did not increase the risk of hypertension in men, regular RT is also recommended for men to improve their musculoskeletal fitness and health.

Lifestyle modifications, including regular aerobic exercise training, are recommended for preventing and/or managing hypertension. However, the antihypertensive effects of RT are controversial. A recent cohort study revealed that engaging in RT for more than 1 day/week decreased the risk of hypertension by 19% in an Australian cohort (15). However, the study did not consider potential sex-based differences in the antihypertensive effects of RT. Accordingly, we investigated sex-based differences in the association between RT regularity and hypertension risk reduction in Korea. In the present study, although men had significantly higher RT levels including training time, period, and frequency, than women, performing RT was associated with a reduction in hypertension by 8% in women but not in men. This is consistent with the results of a recent meta-analysis that reported that regular RT significantly reduced both SBP and DBP in women but not in men (11). Although the potential mechanism underlying sex-based differences in the antihypertensive effect of RT has not been fully elucidated, RT-related changes in arterial stiffness may be the cause of this effect. According to prospective studies, a higher level of arterial stiffness in normotensive participants is an independent predictor of new-onset hypertension, as well as increased BP (22, 23). In a previous study, neither moderate- nor high-intensity long-term RT programs increased arterial stiffness in women (24). Another RCT reported that moderate-intensity RT for 16 weeks significantly improved arterial stiffness in women (25). In contrast, men who had been performing moderate-to-high-intensity long-term RT programs had higher arterial stiffness and SBP than those had by the control group (26). In another study, although 4 weeks of moderate-intensity RT reduced DBP and did not increase arterial stiffness in women, a significant increase in arterial stiffness was observed in men after they followed the same RT program (27). Therefore, based on previous studies, RT-related changes in arterial stiffness are likely due to sex-based differences in the antihypertensive effects of RT. However, according to recent studies, higher levels of muscular strength, which is a major outcome of long-term RT, are significantly related to a reduction in the risk of hypertension in both sexes (18, 19). Given these contradictory findings, sex-based differences in the antihypertensive effects of RT have not been fully investigated. The possible mechanisms underlying these differences in RT-related changes in arterial stiffness remain unclear. Further prospective studies that simultaneously consider both participation in RT and long-term changes in arterial stiffness are needed to investigate RT-related changes in arterial stiffness, which could explain sex-based differences in the risk of incident hypertension beyond the BP-lowering effect.

Current guidelines recommend engaging in RT for 2–3 days/week to improve BP as well as musculoskeletal fitness (5, 6, 9). However, to our knowledge, few studies have investigated whether there is an inverse-graded dose–response relationship between RT frequency and the risk of hypertension. Although a recent meta-analysis has demonstrated that significant BP-lowering effects were observed with 3 days/week of RT compared with 2 days/week of RT (11), additional effects of RT at frequencies exceeding the current guidelines were not evaluated. Accordingly, we further examined the presence of an inversely graded dose–response relationship between RT frequency and the risk of hypertension. Our findings showed no inverse dose–response relationship between RT frequency and the risk of hypertension in either sex, and no significant association between any of the training frequencies and the risk of hypertension was observed in men. In contrast, the risk of hypertension was reduced by 11% in women who performed RT for 3–4 days per week, even after adjusting for covariates, including the total training volume (e.g., RT time per week and period). Our findings are consistent with those of previous studies. In recent RCTs, no significant reductions in both SBP and DBP were observed in middle-aged women after a long-term RT program (≥12 weeks) for 2 days/week at moderate- (28) and progressive-intensity (from moderate- to high-intensity) levels, regardless of their menopausal status (29, 30). In contrast, previous RCTs have reported significant BP-lowering effects in postmenopausal women following a 12-week RT program for 3 days/week at low- (31), moderate- (32), and progressive- intensity (from low- to moderate-intensity) levels (33). Another RCT also reported a significant BP-lowering effect in an obese population that was mostly women (more than 70% of the total participants) after 24 weeks of progressive (from moderate-to-high-intensity) RT for 4 days/week (34). Similar findings have been reported for the effect of different weekly frequencies of RT on musculoskeletal fitness and body composition. After 12 weeks of a high-intensity RT program, body fat percentage significantly decreased in the group that performed RT for 4 days/week but not in the group that performed RT for 2 days/week (35). After 8 weeks of a high-intensity RT program, even when the total training volume was consistent per week, performing RT for 4 days/week provided a greater increase in muscular strength than that observed with a frequency of 2 days/week (36). Our findings and those of previous studies suggest that performing RT for 3–4 days/week is recommended to prevent hypertension and improve muscular fitness in women. Considering that RT did not increase the risk of hypertension in men, regular RT for at least 3–4 days/week is recommended to improve musculoskeletal fitness and body composition in men.

We also evaluated the risk of hypertension by simultaneously considering both the RT frequency and period. When RT was performed for at least 6 months, RT frequencies of 3–4 days/week and ≥5 days/week were significantly related to 14 and 11% hypertension risk reduction, respectively, in only women. Notably, even when RT was performed for ≥6 months, no inverse dose–response relationship between RT frequency and the risk of hypertension in either sex was observed. A meta-analysis demonstrated that short-term RT (≤6 weeks) had no effect on BP, while long-term RT for more than 24 weeks (6 months) markedly reduced SBP and DBP by 5.1 and 4.9 mmHg, respectively (37). According to another study that recruited older adult women, long-term RT for more than 8 months compared with a sedentary condition was significantly related to lower SBP and circulating levels of proinflammatory cytokines, such as tumor necrosis factor alpha and interleukin 6 (38). Accordingly, these results indicate that long-term RT for at least 6 months is recommended to manage and/or prevent hypertension. The potential mechanisms underlying the reduction in BP after regular RT include increased endothelial function and vascular conductance. According to RCTs, endothelial function, measured as brachial artery flow-mediated dilation, and plasma levels of nitric oxide metabolites significantly improved after 12 weeks of an RT program (39–41). Furthermore, long-term RT for 6 months markedly increased the resting diameter of the brachial artery and decreased its wall-to-lumen ratio, beyond improving endothelial function (42). Therefore, regular RT may be associated with protective benefits against hypertension by improving vasodilatory function, and if RT is performed for longer periods, it may even cause structural adaptations in the conduit arteries. However, the training intensity of long-term RT, which was not considered in our study and the previous study (42), may play an important role in the antihypertensive effects of RT. Since high-intensity RT and the concurrent Valsalva maneuver are likely to increase central arterial stiffness by increasing plasma norepinephrine levels and BP during exercise (43), further RCTs are required to verify the mechanism behind RT-related antihypertensive effects by simultaneously considering training intensity and changes in vascular structure and function.

One of the crucial strengths of our study is the use of large nationwide cohorts representative of the general Korean population aged 40–79 years. However, this study had some limitations. First, because we only included the Korean population, our findings may not be applicable to other populations. Second, we were unable to deduce cause-and-effect associations due to the cross-sectional design of our study. Third, a self-report questionnaire was used to assess RT levels, which may have introduced recall bias. Finally, specific information on RT intensity was not available from the self-reported questionnaire. Further studies are required to verify the optimal frequency, intensity, type, volume, and training period of RT for the management and/or prevention of hypertension.

In conclusion, our findings show that when RT was performed for at least 6 months, training frequencies of 3–4 days/week and ≥5 days/week were significantly associated with 14 and 11% hypertension risk reduction, respectively, in women only. Furthermore, as no inverse dose–response association between RT frequency and hypertension risk was observed, engaging in RT for 3–4 days/week for at least 6 months is recommended for women. Considering that RT did not increase the risk of hypertension in men, regular RT is also recommended for men to improve musculoskeletal fitness and health. However, it is important to note the cross-sectional design of the present study; further longitudinal studies are required to validate our findings.

## Data availability statement

Publicly available datasets were analyzed in this study. This data can be found here: the Korean Genome and Epidemiology Study (KoGES; 6635-302), Korea National Institute of Health, Korea Disease Control and Prevention Agency.

## Ethics statement

The studies involving humans were approved by the Institutional Review Board of the Korea National Institute of Health, Korea Disease Control and Prevention Agency (Approval No. KDCA-2024-02-12-P-01). The studies were conducted in accordance with the local legislation and institutional requirements. The participants provided their written informed consent to participate in this study.

## Author contributions

JHP: Writing – review & editing, Writing – original draft, Visualization, Validation, Software, Investigation, Formal analysis, Conceptualization. H-YP: Writing – review & editing, Supervision, Resources, Project administration, Methodology, Funding acquisition, Data curation, Conceptualization.
