# Supplementary material for: Sex-based differences in the association of resistance training levels with the risk of hypertension
Source: Front Public Health. 2024 Jun 6;12:1401254. doi: 10.3389/fpubh.2024.1401254 (PMC11187993; doi:10.3389/fpubh.2024.1401254)
Supplement: Supplementary file 1 [file Table_1.DOCX]

**Supplementary Table 1.** Characteristics of study participants based on hypertension status and sex

| **Variables** | **Men** (n = 56,282) | | ***p*-value** | **Women** (n = 105,820) | | ***p*-value** |
| --- | --- | --- | --- | --- | --- | --- |
|  | **Normotensive**  (n = 35,864) | **Hypertension**  (n = 20,418) |  | **Normotensive**  (n = 77,315) | **Hypertension**  (n = 28,505) |  |
| **Age** (years) | 52.48 ± 8.54 | 56.42 ± 8.55 | < 0.0001 | 51.08 ± 7.70 | 57.16 ± 7.73 | < 0.0001 |
| **Educational level**, n (%) |  |  | < 0.0001 |  |  | < 0.0001 |
| ≤Elementary school | 4,057 (11.31) | 3,246 (15.90) |  | 14,635 (18.93) | 10,878 (38.16) |  |
| Middle/high school | 18,886 (52.66) | 11,121 (54.47) |  | 46,741 (60.45) | 15,051 (52.80) |  |
| ≥College | 12,921 (36.03) | 6,051 (29.63) |  | 15,939 (20.62) | 2,576 (9.04) |  |
| **Drinking habit**, n (%) |  |  | < 0.0001 |  |  | < 0.0001 |
| Never drinker | 7,663 (21.37) | 3,571 (17.49) |  | 50,063 (64.75) | 20,327 (71.31) |  |
| Ex-drinker | 2,286 (6.37) | 1,510 (7.40) |  | 1,499 (1.94) | 630 (2.21) |  |
| Current drinker | 25,915 (72.26) | 15,337 (75.11) |  | 25,753 (33.31) | 7,548 (26.48) |  |
| **Smoking habit**, n (%) |  |  | < 0.0001 |  |  | < 0.0001 |
| Never smoker | 9,667 (26.95) | 5,921 (29.00) |  | 74,380 (96.20) | 27,654 (97.02) |  |
| Ex-smoker | 13,280 (37.03) | 8,594 (42.09) |  | 1,019 (1.32) | 292 (1.02) |  |
| Current smoker | 12,917 (36.02) | 5,903 (28.91) |  | 1,916 (2.48) | 559 (1.96) |  |
| **PA time** (min/week) | 160.17 ± 230.91 | 177.08 ± 242.67 | < 0.0001 | 136.96 ± 203.94 | 140.68 ± 208.84 | < 0.01 |
| **RT**, n (%) | 5,702 (15.90) | 3,215 (15.75) | 0.63 | 11,532 (14.92) | 3,116 (10.93) | < 0.0001 |
| **BMI** (kg/m^2^) | 23.94 ± 2.66 | 25.13 ± 2.81 | < 0.0001 | 23.29 ± 2.78 | 25.03 ± 3.16 | < 0.0001 |
| **WC** (cm) | 84.57 ± 7.34 | 87.94 ± 7.52 | < 0.0001 | 77.46 ± 7.88 | 82.56 ± 8.42 | < 0.0001 |
| **SBP** (mmHg) | 119.48 ± 10.76 | 137.04 ± 14.99 | < 0.0001 | 115.50 ± 11.59 | 136.49 ± 15.76 | < 0.0001 |
| **DBP** (mmHg) | 75.14 ± 7.46 | 86.14 ± 10.09 | < 0.0001 | 71.91 ± 7.90 | 83.89 ± 10.02 | < 0.0001 |
| **T-Chol** (mg/dL) | 194.62 ± 33.93 | 194.32 ± 35.59 | 0.32 | 198.88 ± 35.38 | 203.61 ± 36.73 | < 0.0001 |
| **HDL-C** (mg/dL) | 49.46 ± 11.97 | 48.70 ± 12.10 | < 0.0001 | 56.52 ± 12.97 | 53.16 ± 12.65 | < 0.0001 |
| **TG** (mg/dL) | 145.31 ± 103.44 | 169.62 ± 122.11 | < 0.0001 | 107.51 ± 69.72 | 137.05 ± 87.80 | < 0.0001 |
| **FBG** (mg/dL) | 97.05 ± 23.22 | 103.43 ± 27.53 | < 0.0001 | 91.26 ± 17.56 | 98.13 ± 23.41 | < 0.0001 |
| **Creatinine** (mg/dL) | 0.97 ± 0.16 | 1.01 ± 0.29 | < 0.0001 | 0.74 ± 0.15 | 0.77 ± 0.24 | < 0.0001 |
| **eGFR** (ml/min per 1.73 m^2^) | 83.61 ± 13.91 | 80.07 ± 15.23 | < 0.0001 | 85.57 ± 15.46 | 80.62 ± 16.41 | < 0.0001 |
| **Diabetes mellitus**, n (%) | 3,472 (9.68) | 3,763 (18.43) | < 0.0001 | 3,848 (4.98) | 4,213 (14.78) | < 0.0001 |

PA time, total time of regular participation in any sport or exercise to the point of sweating; RT, resistance training; BMI, body mass index; WC, waist circumference; SBP, systolic blood pressure; DBP, diastolic blood pressure; T-Chol, total cholesterol; HDL-C, high-density lipoprotein cholesterol; TG, triglyceride; FBG, fasting blood glucose; eGFR, estimated glomerular filtration rate.
