# Supplementary material for: Sex-based differences in the association of resistance training levels with the risk of hypertension
Source: Front Public Health. 2024 Jun 6;12:1401254. doi: 10.3389/fpubh.2024.1401254 (PMC11187993; doi:10.3389/fpubh.2024.1401254)
Supplement: Supplementary file 2 [file Table_2.DOCX]

**Supplementary Table 2.** Odds ratios for hypertension prevalence according to RT regularity in various male subgroups

| **Subgroups** | **N** | **Hypertension**,  n (%) | **RT regularity** | | **OR** (95% CI)  non-RT vs.  ≥3 days/week & ≥6 months | ***p* for interaction** |
| --- | --- | --- | --- | --- | --- | --- |
|  |  |  | **Hypertension**, n (%) | |  |  |
|  |  |  | **non-RT** | **≥3 days/week & ≥6 months** |  |  |
| **Age** (years) |  |  |  |  |  |  |
| <65 | 45,950 | 15,534 (33.81) | 13,613 (33.74) | 1,921 (34.28) | 0.97 (0.91–1.04) | < 0.05 |
| ≥65 | 7,808 | 4,037 (51.70) | 3,590 (51.15) | 447 (56.65) | 1.13 (0.96–1.32) |  |
| **Educational level** |  |  |  |  |  |  |
| ≤Middle school | 14,597 | 6,211 (42.55) | 5,780 (42.48) | 431 (43.58) | 0.98 (0.85–1.12) | 0.89 |
| ≥High school | 39,161 | 13,360 (34.12) | 11,423 (33.84) | 1,937 (35.84) | 1.00 (0.94–1.07) |  |
| **Current drinking habit** |  |  |  |  |  |  |
| No | 14,474 | 4,917 (33.97) | 4,373 (33.84) | 544 (35.03) | 1.05 (0.93–1.18) | 0.21 |
| Yes | 39,284 | 14,654 (37.30) | 12,830 (37.25) | 1,824 (37.69) | 1.00 (0.93–1.07) |  |
| **Smoking status** |  |  |  |  |  |  |
| Never | 35,650 | 13,886 (38.95) | 11,998 (38.99) | 1,888 (38.70) | 1.00 (0.94–1.07) | 0.80 |
| Ever | 18,108 | 5,685 (31.39) | 5,205 (31.37) | 480 (31.68) | 1.00 (0.88–1.13) |  |
| **BMI** (kg/m^2^) |  |  |  |  |  |  |
| <25 | 32,494 | 9,699 (29.85) | 8,598 (29.87) | 1,101 (29.71) | 0.99 (0.92–1.08) | 0.70 |
| ≥25 | 21,264 | 9,872 (46.43) | 8,605 (46.32) | 1,267 (47.15) | 1.03 (0.95–1.13) |  |
| **Diabetes mellitus** |  |  |  |  |  |  |
| No | 46,768 | 15,942 (34.09) | 13,975 (33.96) | 1,967 (35.04) | 1.01 (0.95–1.08) | 0.69 |
| Yes | 6,990 | 3,629 (51.92) | 3,228 (51.98) | 401 (51.41) | 0.96 (0.82–1.13) |  |

RT, resistance training; OR, odds ratio; CI, confidence interval; BMI, body mass index; T-Chol, total cholesterol; eGFR, estimated glomerular filtration rate; PA, physical activity; Adjusted for age, drinking, smoking, educational level, BMI, T-Chol, eGFR, PA time, and diabetes mellitus.
