# Supplementary material for: Sex-based differences in the association of resistance training levels with the risk of hypertension
Source: Front Public Health. 2024 Jun 6;12:1401254. doi: 10.3389/fpubh.2024.1401254 (PMC11187993; doi:10.3389/fpubh.2024.1401254)
Supplement: Supplementary file 3 [file Table_3.DOCX]

**Supplementary Table 3.** Odds ratios for the prevalence of hypertension according to RT regularity in various female subgroups

| **Subgroups** | **N** | **Hypertension**,  n (%) | **RT regularity** | | **OR** (95% CI)  non-RT vs.  ≥3 days/week & ≥6 months | ***p* for interaction** |
| --- | --- | --- | --- | --- | --- | --- |
|  |  |  | **Hypertension**, n (%) | |  |  |
|  |  |  | **non-RT** | **≥3 days/week & ≥6 months** |  |  |
| **Age** (years) |  |  |  |  |  |  |
| <65 | 90,355 | 21,903 (24.24) | 20,197 (24.74) | 1,706 (19.55) | 0.84 (0.79–0.89)^****^ | 0.06 |
| ≥65 | 10,026 | 5,430 (54.16) | 5,192 (54.41) | 238 (49.28) | 0.93 (0.76–1.12) |  |
| **Educational level** |  |  |  |  |  |  |
| ≤Middle school | 43,134 | 16,410 (38.04) | 15,653 (38.45) | 757 (31.23) | 0.82 (0.74–0.90)^****^ | 0.23 |
| ≥High school | 57,247 | 10,923 (19.08) | 9,736 (19.29) | 1,187 (17.49) | 0.91 (0.84–0.98)^**^ |  |
| **Current drinking habit** |  |  |  |  |  |  |
| No | 69,140 | 20,180 (29.19) | 18,771 (29.70) | 1,409 (23.70) | 0.92 (0.86–0.98)^*^ | < 0.001 |
| Yes | 31,241 | 7,153 (22.90) | 6,618 (23.66) | 535 (16.39) | 0.80 (0.72–0.89)^****^ |  |
| **Smoking status** |  |  |  |  |  |  |
| Never | 97,983 | 26,785 (27.34) | 24,865 (27.96) | 1,920 (21.23) | 0.88 (0.83–0.93)^****^ | 0.47 |
| Ever | 2,398 | 548 (22.85) | 524 (23.48) | 24 (14.46) | 0.82 (0.50–1.33) |  |
| **BMI** (kg/m^2^) |  |  |  |  |  |  |
| <25 | 69,807 | 14,494 (20.76) | 13,362 (21.27) | 1,132 (16.21) | 0.85 (0.79–0.91)^****^ | 0.19 |
| ≥25 | 30,574 | 12,839 (41.99) | 12,027 (42.43) | 812 (36.48) | 0.91 (0.83–1.00) |  |
| **Diabetes mellitus** |  |  |  |  |  |  |
| No | 92,598 | 23,254 (25.11) | 21,534 (25.66) | 1,720 (19.80) | 0.89 (0.83–0.94)^****^ | 0.66 |
| Yes | 7,783 | 4,079 (52.41) | 3,855 (53.08) | 224 (43.08) | 0.80 (0.66–0.97)^*^ |  |

RT, resistance training; OR, odds ratio; CI, confidence interval; BMI, body mass index; T-Chol, total cholesterol; eGFR, estimated glomerular filtration rate; PA, physical activity; ^*^, *p* < 0.05; ^**^, *p* < 0.01; ^****^, *p* < 0.0001; Adjusted for age, drinking, smoking, educational level, BMI, T-Chol, eGFR, PA time, and diabetes mellitus.
